# Access to Medications for Opioid Use Disorder Among Veterans With Homeless Experience in Permanent Supportive Housing

**DOI:** 10.1001/jamanetworkopen.2026.10831

**Published:** 2026-05-05

**Authors:** Michael Hsu, Talia Panadero, Larissa J. Mooney, David Kim, David Lawrence, Anita Yuan, Prabha Siddarth, Sonya Gabrielian

**Affiliations:** 1 Veterans Affairs Greater Los Angeles Health Care, Los Angeles, California; 2 Department of Psychiatry and Biobehavioral Sciences, Semel Institute for Neuroscience and Human Behavior, University of California, Los Angeles; 3 David Geffen School of Medicine, University of California, Los Angeles

## Abstract

**Importance:**

Drug overdose is a leading cause of death among US veterans with homeless experience. Medications for opioid use disorder (MOUD) reduce overdose and all-cause mortality, yet receipt remains low among veterans with homeless experience. Identifying factors associated with MOUD receipt in Department of Veterans Affairs (VA) permanent supportive housing (PSH) can inform strategies to support MOUD implementation and advance health equity.

**Objective:**

To identify demographic, clinical, and service-related factors associated with MOUD receipt among veterans with homeless experience with opioid use disorder (OUD) in the Housing and Urban Development–VA Supportive Housing (HUD-VASH) program.

**Design, Setting, and Participants:**

This cohort study used linked national VA administrative and electronic health record data. US veterans with OUD entering HUD-VASH between October 1, 2017, and September 30, 2021, were followed up for 12 months after move-in. Eligible participants were diagnosed with OUD as defined by *International Statistical Classification of Diseases and Related Health Problems, Tenth Revision* criteria within 5 years before or 1 year after move-in. Sequential logistic regression models examined associations between demographic, clinical, and service utilization factors and MOUD receipt. Statistical analyses were conducted between May 2025 and February 2026.

**Exposure:**

Demographic, clinical, and service-related factors.

**Main Outcomes and Measures:**

Receipt of MOUD (buprenorphine, methadone, or extended-release naltrexone) within 12 months after HUD VASH move-in.

**Results:**

Among 10 110 veterans with OUD, the mean (SD) age was 53.2 (12.0) years (9297 male [92%]; 3211 Black [32%], 606 Hispanic [6%], 5537 White [55%]); 1685 veterans (17%) received MOUD. Older age (age 55 to 64 years: adjusted odds ratio [AOR], 0.52 [0.42-0.64]) and non-Hispanic Black race (AOR, 0.47 [95% CI, 0.40-0.55]) were associated with lower odds of MOUD receipt, while depression (AOR, 1.24 [95% CI, 1.05-1.46]) and greater behavioral health engagement was associated with higher odds of receipt. Having 1 or more instances of inpatient hospitalization was associated with lower odds of MOUD receipt (AOR, 0.74 [95% CI, 0.64-0.87]).

**Conclusions and Relevance:**

In this cohort study of veterans with homeless experience with OUD, 1 in 6 veterans received MOUD within a year of entering PSH. Lower rates of MOUD receipt among older veterans and veterans from racial minority groups highlighted persistent inequities, regardless of housing status.

## Key Points

**Question:**

Among veterans with homeless experience with opioid use disorder (OUD) residing in permanent supportive housing (PSH), what factors are associated with receipt of medications for opioid use disorder (MOUD)?

**Findings:**

In a cohort study of 10 110 US veterans entering a federal supportive housing program, only 17% received MOUD within 12 months. Greater behavioral health engagement was associated with MOUD receipt, while older age, race minority status, and prior inpatient hospitalization were associated with lower odds.

**Meaning:**

These results suggest that MOUD access in supportive housing should be expanding, which could require embedding addiction care into PSH teams, leveraging behavioral health touchpoints, and addressing persistent disparities.

## Teaser

This cohort study of US veterans with opioid use disorder (OUD) in a federal housing support program examines the association of socioeconomic and clinical characteristics with receipt of OUD medication.

## Introduction

Drug overdose is a leading cause of death among veterans with homeless experience in the US.^1^ Despite the proven effectiveness of medications for opioid use disorder (MOUD) in reducing overdose and opioid-related mortality, access to these treatments remains low among veterans with homeless experience.^2^ Identifying strategies to increase MOUD uptake may decrease morbidity and mortality in a population that faces higher risks.

Three MOUD are approved by the US Food and Drug Administration: buprenorphine, methadone, and naltrexone. Buprenorphine and methadone have consistently been shown to reduce overdose deaths and all-cause mortality, improve treatment retention, and enhance quality of life among individuals with opioid use disorder (OUD).^2,3^ However, many structural and contextual barriers—such as fragmented care, transportation difficulties, and stigma—limit access to MOUD, particularly among persons with homeless experience.^4,5,6^ Health systems that integrate housing and health services are uniquely positioned to address these barriers and test strategies that increase MOUD access.

The US Department of Veterans Affairs (VA) operates the nation’s largest integrated health system and is the largest single provider of permanent supportive housing (PSH) through the Housing and Urban Development–VA Supportive Housing (HUD-VASH) program. HUD-VASH is central to VA’s strategic plan to end veteran homelessness and combines rent subsidies with field-based case management and supportive services, including nonmandated linkages to health care. Although HUD-VASH has markedly reduced homelessness among veterans, it has not significantly improved substance use disorder (SUD) outcomes or overdose mortality.^7,8^ Implementing and sustaining evidence-based interventions for SUD and overdose prevention within PSH settings remains a critical unmet need.

Increasing MOUD use among veterans with homeless experience in PSH would not only save lives but advance equity for a group marginalized by intersecting vulnerabilities related to housing instability, substance use, and race and ethnicity. National civilian data have demonstrated persistent racial and ethnic disparities in MOUD receipt, with White and privately insured individuals more likely to receive buprenorphine than their racial and ethnic minority or publicly insured counterparts.^4,6,9^ To address access gaps, federal and VA policies expanded access to buprenorphine through telehealth initiation and relaxed in-person requirements during the COVID-19 pandemic. However, factors associated with MOUD receipt—and potential disparities—among veterans with homeless experience residing in PSH remain unexamined. This study explored demographic, clinical, and service-related factors associated with MOUD receipt among veterans with homeless experience with OUD in PSH. Findings may inform data-driven strategies to promote equitable access to MOUD and reduce overdose mortality.

## Methods

### Data and Sample

We obtained national VA data from administrative records from Homeless Management Evaluation System (HOMES) and electronic health record data from the VA Corporate Data Warehouse (CDW). HOMES is an administrative database maintained by VA homeless services staff and was used to obtain information on participation in VA homeless programs, including HUD-VASH. CDW was used to obtain information on demographics, medication prescriptions, and health care utilization. VA CDW data were available from October 1, 2013, through September 30, 2022.

We identified a cohort of 10 124 veterans who enrolled in the HUD-VASH program between October 1, 2017, and September 30, 2021 (from HOMES), and had a diagnosis of OUD based on *International Statistical Classification of Diseases and Related Health Problems, Tenth Revision (ICD-10)* codes in the 5 years prior to and/or 1 year following HUD-VASH move-in (from CDW). The study period began in fiscal year 2018 based on data availability and the analytic timeframe for this operational quality improvement project, allowing for sufficient longitudinal follow-up using the most recent complete VA data (fiscal years 2018-2021). Of the 10 124 veterans with OUD enrolled in HUD-VASH, 14 were excluded due to missing demographic data (2 missing gender and 12 missing age), resulting in an analytic sample of 10 110 veterans. Study activities were deemed nonresearch by the VA Greater Los Angeles institutional review board. This study complies with Strengthening the Reporting of Observational Studies in Epidemiology (STROBE) reporting guideline for cohort studies.

### Measures

We extracted veteran demographics from CDW, including age at HUD-VASH entry, race, ethnicity, gender, marital status, service-connected disability status at HUD-VASH move-in, and urban vs rural residence, based on the veteran’s home address. Diagnoses were measured using methods developed by the Program Evaluation and Resource Center (PERC)—VA’s national mental health evaluation service—to identify mental health and SUD diagnoses based on *ICD-10* codes and the CDW dataset (eTable 1 in Supplement 1). Per guidance from VA operational partners, we used *ICD-10* codes from the 5 years before and 1 year after HUD-VASH move-in to capture chronic and recent diagnoses. A 5-year lookback period was selected to comprehensively characterize diagnostic information among veterans with homeless experience prior to HUD-VASH move-in, with the goal of capturing chronic conditions that may have been diagnosed or treated several years prior to HUD-VASH engagement. Multiyear lookback periods are commonly used to capture chronic illness diagnoses in electronic health records.^10,11^ We limited follow-up to 1 year after HUD-VASH move-in because this period represents a timeframe of high vulnerability with regards to housing retention, and a 1-year follow-up is common in evaluations of PSH to assess early posthousing outcomes.^12,13^ We identified OUD using *ICD-10* F11.1 (opioid abuse) and F11.2 (opioid dependence) codes, explicitly including “in remission” subcodes (eg, F11.11, F11.21). We included diagnoses in remission because *ICD-10* and *Diagnostic and Statistical Manual of Mental Disorders* (Fifth Edition) recognize remission as a course specifier for people who previously met OUD criteria but currently do not; these patients still carry an OUD diagnosis and remain clinically relevant for access, equity, and relapse-prevention analyses. Excluding in remission codes would systematically omit individuals in sustained recovery, including those whose stability may be due to ongoing MOUD.^14,15^

We also characterized Elixhauser Comorbidity Index for all participants, which identifies the presence of major chronic conditions (eg, diabetes, hypertension) and summarizes them into a single measure of overall comorbidity burden.^16^ We calculated Elixhauser Comorbidity Index scores using *ICD-10* codes from CDW. To avoid potential confounding and double counting with variables included in the study model (eg, depression), we excluded behavioral health diagnoses from the calculation, resulting in scores based on 27 conditions.

#### Care Utilization

We also used CDW to measure VA health care utilization across multiple domains using encounter-level data on visit type, location, and specialty, in the 1 year following HUD-VASH move-in. SUD treatment utilization was defined as having at least 1 documented outpatient visit with the SUD specialty clinic. Emergency department (ED) visits were categorized as low (0-1 visit), middle (2-3 visits), and high (4 or more visits). Primary care and outpatient mental health visit counts were categorized into tertiles (tertile 1, 0-1 visit; tertile 2, 2-3 visits; tertile 3, 4 or more visits). Inpatient admissions were coded as a binary variable indicating at least 1 hospitalization within 1 year of HUD-VASH move-in to any of the following services: acute medicine, spinal cord injury, surgery, psychiatry, SUD (eg, complicated alcohol withdrawal), or intermediary medicine.

#### MOUD

We used CDW data to extract the primary outcome: a binary indicator of whether a veteran filled at least 1 prescription for MOUD within 1 year following HUD-VASH move-in. MOUD included buprenorphine, methadone (from VA-based opioid treatment programs [OTPs]), and extended-release naltrexone. Oral naltrexone was not considered MOUD because it is not typically used nor recommended for OUD due to poor adherence,^17^ and is commonly used to treat alcohol use disorder (AUD), a highly prevalent comorbidity in our study sample.

### Statistical Analyses

Covariates were selected a priori based on the Gelberg-Andersen Behavioral Model for Vulnerable Populations,^18^ including predisposing (demographic), enabling (health care utilization), and need (clinical comorbidity) factors associated with MOUD receipt. First, we conducted descriptive statistics regarding demographics, diagnoses, service utilization, and rates of use for each MOUD. For covariates with minimal missingness (less than 1% for all variables), missing values were recoded to the modal category (marital status to previously married [62 participants]; service connection to no connection [3 participants]; urban/rural status to urban [31 participants]) to preserve sample size. Veterans who identified as Hispanic were categorized as Hispanic regardless of reported race. Among those who were not Hispanic, race was categorized as non-Hispanic Black, non-Hispanic White, or non-Hispanic other. Due to small sample sizes, American Indian or Alaska Native, Asian, Native Hawaiian or Other Pacific Islander, and multiracial veterans were combined into the non-Hispanic other category. Veterans with missing or unknown race or ethnicity were categorized as unknown race and ethnicity.

Second, we used a sequential modeling approach to examine how associations between veteran characteristics and MOUD receipt changed as additional domains of covariates were introduced. Model 1 included demographic variables; model 1A additionally included move-in date on or after March 1, 2020 (during the COVID-19 pandemic); model 2 added clinical diagnoses and comorbidity burden; and model 3 incorporated service utilization variables. Adjusted odds ratios (AORs) and 95% CIs were calculated. Statistical significance was defined as *P* < .05 in 2-sided tests. All statistical analyses were performed using SAS Enterprise Guide version 9.4 (SAS Institute Inc). Statistical analyses were conducted between May 2025 and February 2026.

## Results

### Sample Characteristics

Among 10 110 veterans with OUD in HUD-VASH, the mean (SD) age was 53.2 (12.0) years (9297 male [92%]; 3211 Black [32%], 606 Hispanic [6%], 5537 White [55%]) (Table 1). A total of 1685 veterans (17%) received MOUD within 1 year of HUD-VASH move-in. Of those receiving MOUD, buprenorphine was most common (1571 [93%]) followed by methadone (79 [5%]), and extended-release naltrexone (53 [3%]). Veterans who received MOUD were, on average, younger than those who did not (mean [SD] age, 49.1 [12.8] years vs 54.0 [11.6] years). Common psychiatric conditions included depressive disorder (7930 [78%]), anxiety disorder (6027 [60%]), and posttraumatic stress disorder (PTSD) (5218 [52%]); 8244 participants (82%) had co-occurring AUD while 6848 (68%) had stimulant use disorder.

**Table 1.** Characteristics of Veterans With Opioid Use Disorder in Housing and Urban Development–VA Supportive Housing

Sample characteristics	Veterans, No. (%)		
	Total (N = 10 110)	Receiving MOUD (n = 1685)	No MOUD (n = 8425)
Gender			
Male	9297 (92)	1519 (90)	7778 (92)
Female	813 (8)	166 (10)	647 (8)
Age, y			
Mean (SD)	53.2 (12.0)	49.1 (12.8)	54.0 (11.6)
18-34	1071 (11)	306 (18)	775 (9)
35-44	1514 (15)	377 (22)	1137 (14)
45-54	1663 (16)	269 (16)	1394 (17)
55-64	4430 (44)	563 (33)	3867 (46)
≥65	1432 (14)	170 (10)	1262 (15)
Race and ethnicity			
Hispanic	606 (6)	109 (6)	497 (7)
Non-Hispanic Black	3211 (32)	286 (17)	2925 (35)
Non-Hispanic White	5537 (55)	1158 (69)	4379 (52)
Non-Hispanic other^a^	512 (5)	83 (5)	429 (5)
Unknown race or ethnicity	244 (2)	49 (3)	195 (2)
Marital status			
Married	1145 (11)	218 (13)	927 (11)
Never married	3126 (31)	533 (32)	2593 (31)
Previously married	5839 (58)	934 (55)	4905 (58)
Move-in date relative to COVID-19 pandemic			
Before March 1, 2020	6858 (68)	1107 (66)	5751 (68)
On or after March 1, 2020	3252 (32)	578 (34)	2674 (32)
Service connected (yes)^b^	5907 (58)	1100 (65)	4807 (57)
Urban or rurality			
Urban	8686 (86)	1416 (84)	7269 (86)
Rural	1408 (14)	269 (16)	1139 (14)
Missing	31 (<1)	3 (<1)	28 (<1)
Health service utilization 1-y after move-in			
≥1 Health care visit^c^	9601 (95)	1679 (>99)	7922 (94)
≥1 SUD specialty visit	5053 (50)	1465 (87)	3588 (43)
≥1 Inpatient visit	2963 (29)	548 (33)	2415 (29)
Emergency department utilization			
Low utilization (0 visits)	4565 (45)	673 (40)	3892 (46)
Middle utilization (1-3 visits)	3776 (37)	688 (41)	3088 (37)
High utilization (≥4 visits)	1769 (18)	324 (19)	1445 (17)
Primary care utilization			
Tertile 1 (0-1 visits)	3835 (38)	631 (37)	3204 (38)
Tertile 2 (2-3 visits)	2691 (27)	427 (25)	2264 (27)
Tertile 3 (≥3 visits)	3584 (35)	627 (37)	2957 (35)
Mental health care utilization			
Tertile 1 (0-3 visits)	3465 (34)	149 (9)	3316 (39)
Tertile 2 (4-23 visits)	3265 (32)	620 (37)	2645 (31)
Tertile 3 (≥24 visits)	3380 (33)	919 (54)	2464 (29)
Mental health, SUD, and other diagnoses			
Mental health diagnoses			
Depressive disorders	7930 (78)	1400 (83)	6530 (78)
Bipolar disorder	2656 (26)	443 (26)	2213 (26)
Anxiety disorders	6027 (60)	1144 (68)	4883 (58)
PTSD	5218 (52)	958 (57)	4260 (51)
Psychotic disorders	1407 (14)	184 (11)	1223 (15)
Personality disorders	2148 (21)	335 (20)	1813 (22)
Dementia	146 (1)	17 (1)	129 (2)
SUD diagnoses			
Alcohol use disorder	8244 (82)	1341 (80)	6903 (82)
Stimulant use disorder	6848 (68)	1148 (68)	5700 (68)
Cannabis use disorder	4997 (49)	833 (49)	4164 (49)
Sedative or hypnotic use disorder	1542 (15)	407 (24)	1135 (13)
Inhalant use disorder	109 (1)	19 (1)	90 (1)
Elixhauser, mean (SD)^d^	10.6 (10.8)	11.3 (11.0)	10.5 (10.7)
Tertile 1 (−8 to 3)	3371 (33)	524 (31)	2847 (34)
Tertile 2 (3 to 13)	3226 (32)	535 (32)	2691 (32)
Tertile 3 (≥14)	3513 (35)	626 (37)	2887 (34)

Abbreviations: MOUD, medications for opioid use disorder; PTSD, posttraumatic stress disorder; SUD, substance use disorder; VA, Department of Veterans Affairs.

^a^ Non-Hispanic other category includes American Indian or Alaska Native, Asian, Native Hawaiian or Other Pacific Islander, and multiracial veterans.

^b^ Service connected refers to veterans with a VA-determined disability related to military service, which confers eligibility for enhanced benefits and priority access to care.

^c^ Includes inpatient, emergency department, primary care physician, or mental health visit within the VA.

^d^ Elixhauser Comorbidity Index Score is a measure of overall severity of comorbidities. The higher the score, the higher the comorbidities. In this study analysis, the substance use and mental health diagnoses were removed from Elixhauser calculations to avoid overadjusting for SUDs and mental health diagnoses.

### Factors Associated With MOUD Receipt

In the fully adjusted model, older age was associated with lower odds of MOUD receipt (Table 2). Non-Hispanic Black veterans (AOR, 0.47 [95% CI, 0.40-0.55]) and those who were categorized as non-Hispanic other (AOR, 0.75 [95% CI, 0.58-0.99]) had less odds of receiving MOUD compared with non-Hispanic White veterans. A HUD-VASH move-in date on or after March 1, 2020, was associated with increased odds of MOUD receipt (AOR, 1.38 [95% CI, 1.21-1.56]) compared with a move-in date before March 1.

**Table 2.** Factors Associated With Medications for Opioid Use Disorder Receipt Among Veterans in Housing and Urban Development–VA Supportive Housing, Step-Wise Models

Characteristics	OR (95% CI)			
	Model 1 (demographics)	Model 1A (demographics and move-in date relative to COVID-19 pandemic)	Model 2 (demographics, move-in date, and diagnoses)	Model 3 (demographics, move-in date, diagnoses, and service utilization)
**Demographics**				
Gender				
Male	1 [Reference]	1 [Reference]	1 [Reference]	1 [Reference]
Female	0.98 (0.82-1.18)	0.98 (0.82-1.18)	1.00 (0.82-1.21)	1.01 (0.81-1.24)
Age, y				
18-34	1 [Reference]	1 [Reference]	1 [Reference]	1 [Reference]
35-44	0.81 (0.68-0.97)	0.81 (0.67-0.96)	0.80 (0.67-0.96)	0.81 (0.66-0.99)
45-54	0.52 (0.43-0.63)	0.52 (0.43-0.63)	0.51 (0.42-0.62)	0.55 (0.44-0.68)
55-64	0.45 (0.38-0.53)	0.44 (0.37-0.53)	0.41 (0.34-0.49)	0.52 (0.42-0.64)
≥65	0.43 (0.35-0.54)	0.425 (0.34-0.53)	0.37 (0.29-0.47)	0.57 (0.43-0.74)
Race and ethnicity				
Non-Hispanic White	1 [Reference]	1 [Reference]	1 [Reference]	1 [Reference]
Hispanic	0.82 (0.66-1.02)	0.82 (0.66-1.01)	0.87 (0.69-1.08)	0.91 (0.71-1.16)
Non-Hispanic Black	0.44 (0.38-0.51)	0.44 (0.38-0.51)	0.48 (0.41-0.56)	0.47 (0.40-0.55)
Non-Hispanic other^a^	0.74 (0.58-0.94)	0.74 (0.58-0.94)	0.79 (0.62-1.01)	0.75 (0.58-0.99)
Unknown	0.95 (0.69-1.31)	0.94 (0.68-1.30)	0.94 (0.68-1.30)	0.91 (0.64-1.29)
Marital status				
Never married	1 [Reference]	1 [Reference]	1 [Reference]	1 [Reference]
Married	1.11 (0.93-1.30)	1.12 (0.93-1.33)	1.08 (0.90-1.30)	1.17 (0.96-1.43)
Previously married	1.05 (0.93-1.19)	1.055 (0.93-1.19)	1.03 (0.91-1.17)	1.07 (0.93-1.22)
Urban or rural				
Urban	1 [Reference]	1 [Reference]	1 [Reference]	1 [Reference]
Rural	0.98 (0.84-1.13)	0.98 (0.85-1.14)	0.98 (0.85-1.14)	1.03 (0.87-1.21)
Service connected (yes vs no)^b^	1.14 (1.01-1.23)	1.14 (1.01-1.28)	1.12 (0.99-1.26)	1.06 (0.93-1.20)
Move-in date (on or after March 1, 2020 vs before March 1 2020)	NA	1.13 (1.01-1.26)	1.10 (0.98-1.24)	1.38 (1.21-1.56)
**Diagnoses**				
Mental health diagnoses				
Depressive disorders	NA	NA	1.35 (1.16-1.57)	1.24 (1.05-1.46)
Bipolar disorder	NA	NA	0.89 (0.78-1.01)	0.88 (0.77-1.02)
Anxiety disorders	NA	NA	1.10 (0.98-1.25)	1.01 (0.89-1.16)
PTSD	NA	NA	1.05 (0.93-1.18)	1.00 (0.88-1.13)
Psychotic disorders	NA	NA	0.79 (0.66-0.94)	0.86 (0.71-1.03)
Personality disorders	NA	NA	0.80 (0.70-0.93)	0.81 (0.70-0.95)
Demetia	NA	NA	0.76 (0.45-1.28)	0.78 (0.45-1.35)
SUD diagnoses				
Alcohol use disorder	NA	NA	0.80 (0.69-0.93)	0.49 (0.41-0.58)
Cannabis use disorder	NA	NA	0.86 (0.76-0.97)	0.78 (0.69-0.89)
Stimulant use disorder	NA	NA	1.10 (0.97-1.25)	0.81 (0.70-0.93)
Inhalant use disorder	NA	NA	0.80 (0.48-1.34)	0.70 (0.41-1.20)
Sedative/hypnotic use disorder	NA	NA	1.63 (1.42-1.88)	1.49 (1.28-1.73)
Elixhauser score^c^				
Tertile 1	NA	NA	1 [Reference]	1 [Reference]
Tertile 2	NA	NA	1.24 (1.08-1.43)	1.22 (1.05-1.42)
Tertile 3	NA	NA	1.62 (1.40-1.87)	1.54 (1.31-1.80)
**Care utilization**				
≥1 substance use disorder specialty visit	NA	NA	NA	6.38 (5.37-7.57)
≥1 inpatient visit	NA	NA	NA	0.74 (0.64-0.87)
Emergency department visits				
Low utilization	NA	NA	NA	1 [Reference]
Mid utilization	NA	NA	NA	1.02 (0.88-1.17)
High utilization	NA	NA	NA	0.85 (0.69-1.03)
Primary care visits				
Tertile 1	NA	NA	NA	1 [Reference]
Tertile 2	NA	NA	NA	0.83 (0.71-0.97)
Tertile 3	NA	NA	NA	0.92 (0.80-1.07)
Mental health visits				
Tertile 1	NA	NA	NA	1 [Reference]
Tertile 2	NA	NA	NA	3.26 (2.65-4.02)
Tertile 3	NA	NA	NA	4.13 (3.31-5.16)

Abbreviations: NA, not applicable; OR, odds ratio; PTSD, posttraumatic stress disorder; SUD, substance use disorder; VA, Department of Veterans Affairs.

^a^ Non-Hispanic other category includes American Indian or Alaska Native, Asian, Native Hawaiian or Other Pacific Islander, and multiracial veterans.

^b^ Service connected refers to veterans with a VA-determined disability related to military service, which confers eligibility for enhanced benefits and priority access to care.

^c^ Elixhauser Comorbidity Index Score is a measure of overall severity of comorbidities. The higher the score, the higher the comorbidities. In this study analysis, the substance use and mental health diagnoses were removed from Elixhauser calculations to avoid overadjusting for SUDs and mental health diagnoses.

Depressive disorders were associated with higher odds of MOUD receipt (AOR, 1.24 [95% CI, 1.05-1.46]), whereas personality disorders were associated with lower odds (AOR, 0.81 [95% CI, 0.70-0.95]). Co-occurring AUD, cannabis use disorder, and stimulant use disorder were associated with reduced odds of MOUD receipt.

Veterans with at least 1 SUD-related visit had 6-fold higher rates of receiving MOUD (AOR, 6.38 [95% CI, 5.37-7.57]). Frequent mental health visits were also associated with increased odds of MOUD receipt. Having 1 inpatient admission in the year following HUD-VASH move-in was associated with decreased odds of MOUD receipt (AOR, 0.74 [95% CI, 0.64-0.87]). No significant associations were observed with gender, ethnicity, marital status, rurality, or service connection.

### Stratified Analyses by Co-Occurring AUD Status

In sensitivity analyses stratified by co-occurring AUD (eTable 2 in Supplement 1), older age was associated with lower MOUD receipt among veterans with co-occurring OUD and AUD but not among veterans without co-occurring AUD. Inpatient utilization was associated with lower MOUD receipt among veterans with co-occurring OUD and AUD (AOR, 0.74 [95% CI, 0.63-0.88]) but not among those without co-occurring AUD.

## Discussion

In a national cohort study of 10 110 veterans with OUD enrolled in HUD-VASH between fiscal years 2018 and 2021, 16.7% received MOUD within 1 year of move-in. Using linked VA administrative and electronic health record data, we examined demographic, clinical, and service-related factors associated with MOUD receipt through sequential logistic regression models.

Our finding that older age was associated with a lower likelihood of MOUD receipt is consistent with prior research in both VA^5^ and civilian populations, including individuals without a history of homelessness.^19,20^ Several factors may underlie declining MOUD receipt with age, including clinician concerns regarding polypharmacy, stigma, or misattribution of signs of OUD to normal aging, and limited clinician knowledge regarding OUD management among older adults.^19,21^ Impairment in work or social roles may be less apparent in older adults, particularly among those who have experienced homelessness and may have had fewer vocational pursuits over their lifetime.^21^ Improving rates of MOUD receipt among older adults in PSH may involve enhancing case manager education and referral mechanisms for OUD in older adults; integrating MOUD into primary care and geriatric care settings that treat older adults; and developing tailored screening tools that account for age-related differences in functional impairment.^22^

Black veterans and veterans from other minoritized racial groups (including Asian, American Indian or Alaska Native, Native Hawaiian or other Pacific Islander, and multiple races) had lower odds of receiving MOUD compared with non-Hispanic White veterans, consistent with racial disparities seen in other VA and civilian studies.^9,23,24^ Although overall drug overdose mortality rates have declined in recent years, emerging evidence indicates a continued increase in mortality among Black and Asian veterans with OUD.^25^ Strategies to address these inequities within HUD-VASH may include strengthening cultural competence and implicit bias training for clinicians and case managers, implementing systematic PSH outreach and engagement programs tailored to veterans belonging to a racial minority groups, and embedding measurement and accountability for equity in MOUD prescribing into VA quality improvement initiatives.^26^ Future studies with larger and more diverse samples are needed to better understand mechanisms underlying racial disparities in access to evidence-based treatment.

We observed a dose-response association of mental health service utilization with MOUD receipt. Greater engagement with mental health services likely increases opportunities for OUD screening, diagnosis, and referral to SUD treatment. Notably, we found no association between primary care utilization and MOUD receipt, suggesting potential opportunities for initiation and coordination of treatment in these settings despite national initiatives such as the VA Consortium to Disseminate and Understand Implementation of Opioid Use Disorder Treatment (CONDUIT) and Stepped Care for Opioid Use Disorder, Train the Trainer (SCOUTT) programs,^27^ which have primarily focused on expanding MOUD prescribing within primary care and general Patient Aligned Care Teams. Additional efforts may be needed to adapt and extend similar implementation supports to homeless service programs such as the Homeless Patient Aligned Care Teams. Drawing on the SCOUTT program model, HUD-VASH may benefit from dedicated efforts to support MOUD implementation in its program (eg, training and technical assistance for primary care and mental health clinicians who serve HUD-VASH patients).^24,28^

The negative association between inpatient hospitalization and MOUD receipt highlights a critical missed opportunity to initiate treatment during admissions for medical or substance-related complications, particularly for veterans with homeless experience who may be disengaged from care. These findings underscore the need for health systems to prioritize initiation of MOUD during hospitalization, especially because opioid withdrawal during admission can reduce tolerance and increase overdose risk after discharge. Barriers such as stigma against homeless persons, limited training, and lack of clear follow-up pathways may impede initiation in inpatient settings.^29^

We observed a modest increase in MOUD receipt following the onset of the COVID-19 pandemic. We caution against overinterpretation of this finding, as the absolute difference was small and may reflect broader secular increases in MOUD receipt over time driven by national efforts to expand access and reduce treatment barriers such as waivers of the requirement for in-person evaluation prior to initiating buprenorphine, allowing buprenorphine initiation and continuation via telehealth, and including audio-only visits.^30^ This period also coincided with VA system-level priorities to expand MOUD access, including opioid safety initiatives and implementation efforts to integrate MOUD into nonspecialty care settings.^28^

In our cohort, 16.7% of veterans with OUD received MOUD within 1 year of HUD-VASH move-in, lower than rates reported in an earlier HUD-VASH cohort^13^ and VA implementation initiatives.^29^ Differences may reflect variation in diagnostic inclusion criteria, lookback periods, and MOUD outcome definitions. For example, our study included veterans with *ICD-10* OUD remission codes, consistent with VA Strategic Analytics for Improvement and Learning (SAIL) quality metrics and prior VA studies^14,15^ and identified OUD diagnoses within 5 years prior to HUD-VASH move-in and 1 year after move-in. Higher MOUD receipt reported in the VA SCOUTT initiative^31^ likely reflects its role as an active implementation intervention with dedicated training and facilitation infrastructure, rather than a directly comparable care setting. SCOUTT supports MOUD implementation in general primary care; veterans entering HUD-VASH often face significant social vulnerabilities that distinguish them from their peers, including acute housing instability and competing priorities that may limit early MOUD engagement.

Within the PSH context, addressing gaps in access to MOUD may require embedding addiction-trained clinicians in HUD-VASH or Assertive Community Treatment teams to enable on-site initiation and coordination of MOUD, particularly for veterans disengaged from traditional clinic-based care. Complementary efforts could include providing training and technical assistance to primary care and mental health clinicians, as well as equipping HUD-VASH case managers and peer support specialists with knowledge and tools to identify and refer veterans with OUD. Finally, data-driven initiatives that proactively identify veterans with homeless experience with OUD not receiving MOUD could support targeted outreach, especially in PSH where competing social needs may impede treatment engagement.

### Limitations

This study has several limitations. First, as an observational study, our findings are subject to confounding. Veterans engaged in specialty SUD clinics may have greater OUD severity or treatment readiness—factors not fully captured in administrative data—which could bias associations toward overestimation, although the extent of this bias is difficult to determine. Second, MOUD receipt was identified through prescriptions filled within the VA, which likely underestimates total receipt by excluding medications obtained through non-VA clinicians (eg, methadone from non-VA specialty OTPs). Third, identification of OUD and other clinical characteristics relied on *ICD-10* codes, which are subject to false negatives due to incomplete documentation and false positives through misclassification.^32^ Recent OUD diagnoses observed after HUD-VASH move-in may reflect improved care engagement and enhanced detection following housing stabilization, rather than solely new onset or worsening substance use. Fourth, because this analysis was conducted as part of a program evaluation limited to veterans participating in HUD-VASH, we were unable to compare MOUD receipt with a matched cohort of veterans not enrolled in HUD-VASH.

## Conclusions

In this cohort study, 1 in 6 veterans with homeless experience with OUD received MOUD within 1 year of entering PSH, underscoring major treatment gaps in this high-risk population. Lower rates of MOUD receipt among older veterans and veterans belonging to a racial minority group highlights persisting inequities. Expanding access to MOUD in HUD-VASH requires targeted implementation strategies that integrate addiction care into HUD-VASH and homeless-tailored primary care settings, strengthening cross-disciplinary collaboration, and embedding equity-focused quality improvement within the VA.

## Notes

Supplement 1. eTable 1. Diagnostic codes for mental health and substance use disorders eTable 2. Factors associated with receipt of medications for opioid use disorder (MOUD) among veterans in Housing and Urban Development-Veterans Affairs supportive housing (HUD-VASH), stratified by co-occurring alcohol use disorder (AUD)

Supplement 2. Data Sharing Statement
