# Supplementary material for: Access to Medications for Opioid Use Disorder Among Veterans With Homeless Experience in Permanent Supportive Housing
Source: JAMA Netw Open. 2026 May 5;9(5):e2610831. doi: 10.1001/jamanetworkopen.2026.10831 (PMC13147188; doi:10.1001/jamanetworkopen.2026.10831)
Supplement: Supplement 1. — eTable 1. Diagnostic codes for mental health and substance use disorders eTable 2. Factors associated with receipt of medications for opioid use disorder (MOUD) among veterans in Housing and Urban Development-Veterans Affairs supportive housing (HUD-VASH), stratified by co-occurring alcohol use disorder (AUD) [file jamanetwopen-e2610831-s001.pdf]

Supplemental Online Content

Hsu M, Panadero T, Mooney LJ, et al. Access to medications for opioid use disorder among veterans with homeless experience in permanent supportive housing. *JAMA Netw Open.* 2026;9(5):e2610831. doi:10.1001/jamanetworkopen.2026.10831

eTable 1. Diagnostic codes for mental health and substance use disorders

eTable 2. Factors associated with receipt of medications for opioid use disorder (MOUD) among veterans in Housing and Urban Development-Veterans Affairs supportive housing (HUD-VASH), stratified by co-occurring alcohol use disorder (AUD)

This supplemental material has been provided by the authors to give readers additional information about their work.

© 2026 Hsu M et al. *JAMA Network Open*.

eTable 1. Diagnostic Codes for mental health and substance use disorders.

eTable 1a. Diagnostic codes for mental health disorders.

Diagnosis Category	ICD-10 Codes
**Depressive Disorders**	F32.0; F32.1; F32.2; F32.3; F32.4; F32.5; F32.9; F33.0; F33.1; F33.2; F33.3; F33.40; F33.41; F33.42; F33.8; F32.89; F33.9; F34.1
**Bipolar Disorder**	F30.10; F30.11; F30.12; F30.13; F30.2; F30.3; F30.4; F30.8; F30.9; F31.0; F31.10; F31.11; F31.12; F31.13; F31.2; F31.30; F31.31; F31.32; F31.4; F31.5; F31.60; F31.61; F31.62; F31.63; F31.64; F31.70; F31.71; F31.72; F31.73; F31.74; F31.75; F31.76; F31.77; F31.78; F31.81; F31.89; F31.9
**Anxiety Disorders**	F06.4; F40.00; F40.01; F40.02; F40.10; F40.11; F40.210; F40.218; F40.220; F40.228; F40.230; F40.231; F40.232; F40.233; F40.240; F40.241; F40.242; F40.243; F40.248; F40.290; F40.291; F40.298; F40.8; F40.9; F41.0; F41.1; F41.3; F41.8; F41.9; F42.; F42.2; F42.3; F42.4; F42.8; F42.9; F45.20; F45.21; F45.29
**Posttraumatic Stress Disorder (PTSD)**	F43.10; F43.11; F43.12
**Psychotic Disorders**	F20.0; F20.1; F20.2; F20.3; F20.5; F20.81; F20.89; F20.9; F22.; F23.; F24.; F25.0; F25.1; F25.8; F25.9
**Personality Disorders**	F60.0; F60.1; F60.2; F60.3; F60.4; F60.5; F60.6; F60.7; F60.8; F60.81; F60.89; F60.9
**Dementia**	F00; F00.2; F01; F03; F03.9; F03.90; F03.91; F03.A; F03.B; F03.C; G30.9

eTable 1b. Diagnostic codes for substance use disorders.

Diagnosis Category	ICD-10 Codes
**Alcohol Use Disorder (AUD)**	F10.10; F10.11; F10.120; F10.121; F10.129; F10.14; F10.150; F10.151; F10.159; F10.180; F10.181; F10.182; F10.188; F10.19; F10.20; F10.21; F10.27; F10.280; F10.281; F10.282; F10.288; F10.29

© 2026 Hsu M et al. *JAMA Network Open*.

Diagnosis Category	ICD-10 Codes
**Opioid Use Disorder**	F11.10; F11.11; F11.120; F11.121; F11.122; F11.129; F11.14; F11.150; F11.151; F11.159; F11.181; F11.182; F11.188; F11.19; F11.20; F11.21; F11.220; F11.221; F11.222; F11.229; F11.23; F11.24; F11.250; F11.251; F11.259; F11.281; F11.282; F11.288; F11.29
**Cannabis Use Disorder**	F12.10; F12.11; F12.120; F12.121; F12.122; F12.129; F12.150; F12.151; F12.159; F12.180; F12.188; F12.19; F12.20; F12.21; F12.220; F12.221; F12.222; F12.229; F12.23; F12.250; F12.251; F12.259; F12.280; F12.288; F12.29
**Sedative/Hypnotic/Anxiolytic Use Disorder**	F13.10; F13.11; F13.120; F13.121; F13.129; F13.14; F13.150; F13.151; F13.159; F13.180; F13.181; F13.182; F13.188; F13.19; F13.20; F13.21; F13.220; F13.221; F13.229; F13.230; F13.231; F13.232; F13.239; F13.24; F13.250; F13.251; F13.259; F13.26; F13.27; F13.280; F13.281; F13.282; F13.288; F13.29
**Stimulant Use Disorder**	F14.10; F14.11; F14.120; F14.121; F14.122; F14.129; F14.14; F14.150; F14.151; F14.159; F14.180; F14.181; F14.182; F14.188; F14.19; F14.20; F14.21; F14.220; F14.221; F14.222; F14.229; F14.23; F14.24; F14.250; F14.251; F14.259; F14.280; F14.281; F14.282; F14.288; F14.29; F15.10; F15.11; F15.120; F15.121; F15.122; F15.129; F15.14; F15.150; F15.151; F15.159; F15.180; F15.181; F15.182; F15.188; F15.19; F15.20; F15.21; F15.220; F15.221; F15.222; F15.229; F15.23; F15.24; F15.250; F15.251; F15.259; F15.280; F15.281; F15.282; F15.288; F15.29
**Hallucinogen Use Disorder**	F16.10; F16.11; F16.120; F16.121; F16.122; F16.129; F16.14; F16.150; F16.151; F16.159; F16.180; F16.183; F16.188; F16.19; F16.20; F16.21; F16.220; F16.221; F16.229; F16.24; F16.250; F16.251; F16.259; F16.280; F16.283; F16.288; F16.29
**Inhalant Use Disorder**	F18.10; F18.11; F18.120; F18.121; F18.129; F18.14; F18.150; F18.151; F18.159; F18.17; F18.180; F18.188; F18.19; F18.20; F18.21; F18.220; F18.221; F18.229; F18.24; F18.250; F18.251; F18.259; F18.27; F18.280; F18.288; F18.29

© 2026 Hsu M et al. *JAMA Network Open*.

**eTable 2. Factors Associated with Receipt of Medications for Opioid Use Disorder (MOUD) Among Veterans in Housing and Urban Development-Veterans Affairs Supportive Housing (HUD-VASH), Stratified by Co-occurring Alcohol Use Disorder (AUD)**		
	**Without Co-occurring AUD (n = 1,866)**	**With Co-occurring AUD (n = 8,244)**
**Characteristics**	Odds Ratio (95% Confidence Interval)	
Demographics		
Gender		
Male	*Reference*	
Female	0.74 (0.48, 1.14)	1.1 (0.86, 1.41)
Age		
18-34	*Reference*	
35-44	0.95 (0.57, 1.58)	0.78 (0.63, 0.97)
45-54	0.89 (0.52, 1.52)	0.50 (0.40, 0.64)
55-64	0.85 (0.51, 1.41)	0.47 (0.37, 0.59)
65+	0.77 (0.41, 1.44)	0.53 (0.39, 0.71)
Race/Ethnicity		
Non-Hispanic White	*Reference*	
Non-Hispanic Black	0.56 (0.38, 0.83)	0.44 (0.37, 0.53)
Hispanic	1.00 (0.52, 1.93)	0.89 (0.68, 1.15)
Non-Hispanic Other[a]	1.00 (0.53, 1.91)	0.71 (0.53, 0.96)
Unknown Race/Ethnicity	0.95 (0.41, 2.19)	0.90 (0.60, 1.33)
Marital Status		
Never Married	*Reference*	
Married	1.62 (1.04, 2.53)	1.14 (0.91, 1.43)
Previously Married	1.45 (1.02, 2.06)	1.02 (0.88, 1.18)
Urban/Rural		
Urban	*Reference*	
Rural	1.01 (0.67, 1.50)	1.04 (0.87, 1.25)
Service Connected[b] (Yes vs. No)	0.92 (0.67, 1.26)	1.08 (0.93, 1.25)
Move-In Date (On or After March 1, 2020 vs. Before March 1 2020)	1.51 (1.11, 2.06)	1.35 (1.17, 1.55)
Diagnoses		
Mental Health Diagnoses		
Depressive disorders	1.29 (0.93, 1.80)	1.24 (1.02, 1.51)
Bipolar disorder	0.87 (0.57, 1.31)	0.89 (0.77, 1.03)
Anxiety disorders	1.45 (1.05, 1.99)	0.94 (0.81, 1.09)
Posttraumatic stress disorder (PTSD)	0.85 (0.62, 1.16)	1.03 (0.90, 1.19)
Psychotic disorders	0.64 (0.35, 1.16)	0.89 (0.73, 1.08)
Personality disorders	0.91 (0.56, 1.48)	0.80 (0.68, 0.95)
Demetia	0.20 (0.02, 2.05)	0.85 (0.48, 1.50)

© 2026 Hsu M et al. *JAMA Network Open*.

eTable 2. Factors Associated with Receipt of MOUD Among Veterans in HUD-VASH, Stratified by Co-occurring AUD		
**Characteristics**	**Without Co-occurring AUD (n = 1,866)**	**With Co-occurring AUD (n = 8,244)**
Substance Use Disorder Diagnoses		
Alcohol use disorder	N/A	
Cannabis use disorder	0.99 (0.70, 1.40)	0.751 (0.65, 0.86)
Stimulant use disorder	0.50 (0.36, 0.70)	0.68 (0.39, 1.20)
Inhalant use disorder	0.74 (0.11, 4.81)	0.91 (0.77, 1.07)
Sedative/hypnotic use disorder	0.70 (0.42, 1.16)	1.58 (1.35, 1.85)
Elixhauser Score[c]		
Tertile 1	*Reference*	
Tertile 2	1.08 (0.77, 1.53)	1.25 (1.06, 1.49)
Tertile 3	1.28 (0.86, 1.92)	1.61 (1.35, 1.93)
Care Utilization		
≥1 Substance use disorder specialty visit	10.93 (7.71, 15.48)	5.44 (4.48, 6.62)
≥1 Inpatient visit	0.80 (0.52, 1.21)	0.74 (0.63, 0.88)
Emergency Dept. Visits		
Low Utilization	*Reference*	
Mid Utilization	1.27 (0.91, 1.78)	0.97 (0.82, 1.14)
High Utilization	0.97 (0.57, 1.65)	0.82 (0.66, 1.02)
Primary Care Visits		
Tertile 1	*Reference*	
Tertile 2	1.13 (0.78, 1.63)	0.77 (0.65, 0.91)
Tertile 3	1.03 (0.71, 1.49)	0.88 (0.76, 1.04)
Mental Health Visits		
Tertile 1	*Reference*	
Tertile 2	2.87 (1.96, 4.21)	3.51 (2.71, 4.54)
Tertile 3	3.96 (2.52, 6.21)	4.42 (3.38, 5.78)

[a] Non-Hispanic Other category includes American Indian or Alaska Native, Asian, Native Hawaiian or Other Pacific Islander, and multiracial Veterans

[b] "Service connected" refers to Veterans with a VA-determined disability related to military service, which confers eligibility for enhanced benefits and priority access to care.

[c] Elixhauser Comorbidity Index Score is a measure of overall severity of comorbidities. The higher the score, the higher the comorbidities. In this study analysis, the substance use and mental health diagnoses were removed from Elixhauser calculations to avoid over adjusting for SUDs and mental health diagnoses.

© 2026 Hsu M et al. *JAMA Network Open*.
