# Supplementary material for: Access to Medications for Opioid Use Disorder Among Veterans With Homeless Experience in Permanent Supportive Housing
Source: JAMA Netw Open. 2026 May 5;9(5):e2610831. doi: 10.1001/jamanetworkopen.2026.10831 (PMC13147188; doi:10.1001/jamanetworkopen.2026.10831)
Supplement: Supplement 2. — Data Sharing Statement [file jamanetwopen-e2610831-s002.pdf]

Data Sharing Statement

Hsu. Access to Medications for Opioid Use Disorder Among Veterans Experiencing Homelessness in Permanent Supportive Housing. *JAMA Netw Open*. Published May 05, 2026. doi:10.1001/jamanetworkopen.2026.10831

Data
Data available: No
